# Pollination and Plant Resources Change the Nutritional Quality of Almonds for Human Health

**DOI:** 10.1371/journal.pone.0090082

**Published:** 2014-02-27

**Authors:** Claire Brittain, Claire Kremen, Andrea Garber, Alexandra-Maria Klein

**Affiliations:** 1 Institute of Ecology, Ecosystem Functions, Leuphana University of Lüneburg, Germany and Department of Entomology, University of California Davis, Davis, California, United States of America; 2 Environmental Sciences Policy and Management, University of California, Berkeley, Berkeley, California, United States of America; 3 Division of Adolescent Medicine, University of California San Francisco, San Francisco, California, United States of America; 4 Institute of Ecology, Ecosystem Functions, Leuphana University of Lüneburg, Germany and Chair of Nature Conservation and Landscape Ecology, University of Freiburg, Germany; Nanjing Agricultural University, China

## Abstract

Insect-pollinated crops provide important nutrients for human health. Pollination, water and nutrients available to crops can influence yield, but it is not known if the nutritional value of the crop is also influenced. Almonds are an important source of critical nutrients for human health such as unsaturated fat and vitamin E. We manipulated the pollination of almond trees and the resources available to the trees, to investigate the impact on the nutritional composition of the crop. The pollination treatments were: (a) exclusion of pollinators to initiate self-pollination and (b) hand cross-pollination; the plant resource treatments were: (c) reduced water and (d) no fertilizer. In an orchard in northern California, trees were exposed to a single treatment or a combination of two (one pollination and one resource). Both the fat and vitamin E composition of the nuts were highly influenced by pollination. Lower proportions of oleic to linoleic acid, which are less desirable from both a health and commercial perspective, were produced by the self-pollinated trees. However, higher levels of vitamin E were found in the self-pollinated nuts. In some cases, combined changes in pollination and plant resources sharpened the pollination effects, even when plant resources were not influencing the nutrients as an individual treatment. This study highlights the importance of insects as providers of cross-pollination for fruit quality that can affect human health, and, for the first time, shows that other environmental factors can sharpen the effect of pollination. This contributes to an emerging field of research investigating the complexity of interactions of ecosystem services affecting the nutritional value and commercial quality of crops.

## Introduction

As the global population grows, so does the demand for food [1]. A balanced diet containing a broad spectrum of nutrients is important for human health [2], [3]. Animal-pollinated crop species provide key nutrients valuable for human health, including an estimated 74% of all globally produced lipids and 35–65% of vitamin E [4]. The benefits of animal pollination for crop production have been documented for many crop species [5]–[7], but the effects of animal pollination on the nutritional composition of crops and other measures of quality such as fruit shelf life and therefore its commercial value have just begun to be investigated [8], [9].

Animal pollination may become a limiting resource if the growth of crops reliant on pollination outpaces the growth in the number of honey bee hives [10] and agricultural intensification and habitat loss continue to negatively affect wild bees [11]–[13]. Human population growth and climate change are predicted to increase the strain on the resources required for food production (particularly water) [14]. While the effects of plant resource shortages have been documented on the yield and development of crops [15]–[17], the effects of the availability of water and other plant resources on nutritional composition are largely unknown. How pollination and resource availability both singly and jointly impact a crop’s nutritional composition is thus a pressing question, particularly in light of the growing demand for certain crops based on their purported nutritional value. Almond (*Prunus dulcis* [Mill.] DA Webb) is an example of a commodity where advertising has focused on the potential health benefits of its consumption [18]. Clinical trials have shown almonds to be cardio-protective [19], an effect that is attributed to their high monounsaturated fat content. The primary monounsaturated fatty acid present in California-grown almonds is oleic acid, an omega-9 fatty acid accounting for 58–74% of the total fat content [20]. Almonds also contain linoleic acid, an omega-6 fatty acid, and very small amounts of linolenic, an omega-3 fatty acid [20]. The health effects of omega-6 fatty acids are controversial, with evidence to suggest that the high omega-6 to omega-3 ratio of the Western diet is a contributor to cardiovascular disease, cancer and diabetes [21]. Almonds are also valued for their vitamin E (α-Tocopherol) content as it is an antioxidant which helps protect cell membranes from peroxidative damage [22].

Since almond is a highly pollinator-dependent crop and a large consumer of water and fertilizer [23], its production may be sensitive to fluctuations in these resources. Furthermore, approximately eighty percent of the world’s almonds are produced in California [24], a state where climate change is expected to reduce water availability [25], [26]. The aim of this study was to investigate if pollination and plant resource availability (water and fertilizer) influence the nutritional composition of almonds. We exposed whole almond trees to different pollination treatments (the exclusion of pollinators to allow for self-pollination only and hand cross-pollination with compatible pollen to achieve high levels of cross-pollination) and different resource treatments (reduced water and no fertilizer). We investigated whether self-pollination and reduced plant resources (fertilizer and water), when experienced by whole trees, influenced the nutritional composition of almonds, and if there were interacting effects. Since almond is highly pollinator dependent [23], we hypothesized that the impact of self-pollination on the nuts’ nutritional content would be of similar strength to that of the availability of water and fertilizer, and that pollen and resource availability would have interacting effects.

## Materials and Methods

The study was conducted in 2008, in a 3.2 ha almond orchard in the Sacramento Valley, Northern California (122°2′1.925″W, 38°55′19.372″N, WGS 1984: the owner of the land gave permission to conduct the study at this site). Our study orchard contained trees of the most popular variety for production, Nonpareil, grafted onto peach rootstock (*Prunus persica* (L.) Batsch) in 2005 and planted in 2006 (third leaf planting). Other tree varieties compatible with Nonpareil were located 100–300 meters away, including Mission (100% compatible), Carrion and Wood Colony (50% compatible). Honey bee hives were placed in the orchard during bloom with the eight nearest hives being 300–350 meters away. As part of the grower’s pollination strategy, all hives in the orchard had Padre pollen (100% compatible) placed at the hive entrance to maximize the transport of compatible pollen to the Nonpareil trees.

### Treatments

Whole trees were exposed to different pollination and resource treatments (Table 1). The treatments were assigned randomly to individual trees in the orchard and were replicated five times in adjacent rows (n = 40 experimental trees). Hand-pollination was conducted from 20–28^th^ February. When flowers opened, Padre pollen was applied to the stigmas using small brushes. The pollen had been harvested before bud opening and stored at −20°C and was thawed before immediate use. All open flowers were hand-pollinated every two to three days until approximately 90% of all buds had opened (the last 10% of buds that opened often had deformed or missing parts). The trees exposed to self-pollination were covered with cages (1.5×2.0 m^2^) with a mesh size of 0.8–1.0 mm from the end of January until mid-March when flowering had ceased.

**Table 1:** The different treatments whole almond trees received (5 trees each, for details see methods).

Treatment	Pollination	Fertilizer	Water
NF no fertilizer	open-pollination	none	normal
RW reduced water	open-pollination	normal	reduced
SP self-pollination	exclusion of pollinators	normal	normal
CP cross-pollination	hand cross-pollination	normal	normal
SP+NF	exclusion of pollinators	none	normal
SP+RW	exclusion of pollinators	normal	reduced
CP+NF	hand cross-pollination	none	normal
CP+RW	hand cross-pollination	normal	reduced

For the three months before bloom, trees were irrigated when necessary and no fertilizer was applied. Once flowering began, three out of the four water emitters for each reduced water tree were closed. Reduced water trees received 27 L water every third day, while the other trees received 108 L. No nutrients were applied to the no fertilizer trees during bloom. Once flowering began, the other trees each received the following: 521.6 g nitrate, 344.7 g of potassium, 244.9 g of sulphur, 158.8 g of calcium, 158.8 g of phosphorus, 54.4 g of magnesium, 27.22 g of boron, 27.22 g of iron, 27.22 g of manganese, and zinc, cobalt, molybdenum, and various other micronutrients (amounts given are per tree, for that growing year). All the experimental trees were similar in height and number of main branches.

On 2^nd^ July, 48 fruits were harvested from each experimental tree. Fruits were randomly selected from the main branches (12, 16 or 24 fruits per branch, corresponding to trees with 4, 3 or 2 main branches). The harvested fruits were dried on the ground for seven days (mimicking harvesting practices), with metal cages protecting them from bird and mammal predation. After the seven days, the hulls were removed from the fruits and the shells were cracked. The almonds were placed in a fridge at 4°C prior to nutritional analyses.

### Nutritional Analyses

All nutritional analyses were performed by NP Analytical Laboratories, St. Louis, MO, U.S.A. and followed the Official Methods of Analysis of the Association of Analytical Communities (AOAC). A single analytical sample was a handful of almonds collected from several branches of an individual tree, and each tree within a treatment group was a replicate. Nuts from trees which had received the four single treatments were analyzed for their full nutritional composition (Table 2). Details of the analytical methods used in the nutritional analysis are given in the supporting information S1. Although each treatment group had five tree replicates in the orchard, some of the trees produced insufficient nuts for nutritional analysis so two treatments (no fertilizer and self-pollination) only had four replicate trees.

**Table 2:** The range of nutrients that were quantified in almonds from trees exposed to a single resource or pollination treatment.

Nutrient	Method of quantification	Units
**Vitamin E**		
Alpha-Tocopherol acetate	HPLC with fluorescent detector	IU/100 g
**Fat composition** ¶		
Oleic, linoleic and linolenic fatty acids, monounsaturated fatty acids, polyunsaturated fatty acids, saturated fatty acids, trans fatty acids, total fat	gas chromatography	g/100 g
**Vitamin B**		
B1 thiamine	manual fluorescence	ppm
B2 riboflavin	semi-automated fluorometric	ppm
B3 niacin	turbidimetric microbiological	ppm
**Minerals**		
Calcium, copper, iron, magnesium, manganese, phosphorus, potassium,sodium, zinc	inductively coupled plasma mass spectrometry	ppm
**Sugar profile**		
Fructose, glucose, lactose, maltose, sucrose	high pressure liquid chromatography	percent

¶ Oleic acid is included in the total monounsaturated fatty acids; linoleic and linolenic acid are included in the total polyunsaturated fatty acids.

The trees which received two treatments (one pollination plus one plant resource) were analyzed for fats and vitamin E composition only, since the levels of these nutrients were found to vary most between samples (see supporting information S2) and almonds are valued for these nutrients. The trees which received two treatments (SP+RW, SP+NF, CP+RW, CP+NF) produced sufficient nuts for 4, 3, 5, and 3 replicates respectively.

### Statistical Analyses: Single Treatments

From the full nutritional analysis, 26 nutrients in almonds were quantified (Table 2) for the four main treatment groups (SP, CP, RW, NF). The samples did not contain any lactose, maltose or trans fatty acids so these were removed from all analyses. We performed non-metric multi-dimensional scaling (NMDS with two dimensions) of the nutritional data, to visualize differences in the nutritional composition of the nuts. To test if differences in the nutritional composition were related to the treatment group, permutational multivariate analysis of variance using Bray-Curtis distances was performed (adonis, R package vegan). *P* values were calculated using F-tests based on sequential sums of squares from 999 permutations of the data. Differences in variation of the nutritional composition between treatment groups were tested using Bray-Curtis distances to the treatment group’s centroid. Analysis of variance of the distances was performed, with treatment group as the explanatory variable (betadisper, R package vegan). *P* values were calculated from 999 permutations of model residuals which were used to generate a permutation distribution of F under the null hypothesis of there being no difference in dispersion between the groups.

### Statistical Analyses: Combined Treatments

The fat and vitamin E composition was analyzed for variation between the eight different treatments: SP, CP, RW, NF, SP+RW, SP+NF, CP+RW, CP+NF. An analysis of variance was carried out for each nutrient separately, with the treatment group as the explanatory variable. The proportion oleic to linoleic fatty acids was also tested as it is an indicator of almond kernel quality [27]. Where treatment was significant (*P*≤0.05), Tukey’s HSD (honestly significant difference) was used to compare between treatments.

A linear mixed effect model was run for each nutrient with the treatment as the explanatory variable. We used a restricted likelihood ratio test to determine if the variance of the random effect of orchard row differed from zero (R package RLRsim). Orchard row was not included as a random effect as the test found that for all nutrients, the variance of the orchard rows did not differ significantly from zero (*P*>0.05). All nutrients were tested for correlation.

## Results

Linoleic acid (an omega-6) is the predominant polyunsaturated fatty acid in almonds, so the values for linoleic acid and total polyunsaturated fatty acids were highly correlated (supporting information S3). Similarly, as the predominant monounsaturated fat, levels of oleic acid were correlated with the total amount of monounsaturated fat and total fat. We will therefore present the results for linoleic and oleic acid but not for monounsaturated fatty acids, polyunsaturated fatty acids or total fat. Levels of linolenic acid were below the threshold for accurate quantification (<0.04 g/100 g).

**Figure 1:**
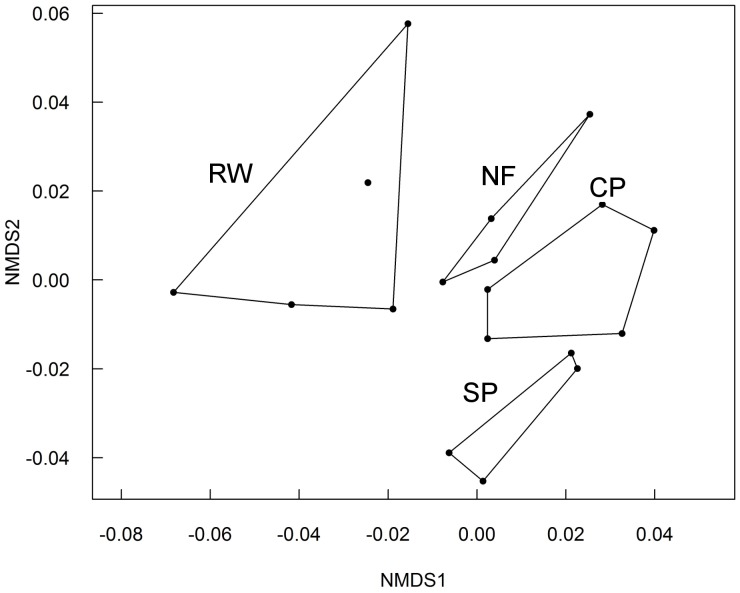
Non-metric multi-dimensional scaling (NMDS) of the nutritional composition of almonds. Almonds were collected from trees that had received one of four treatments: reduced water (RW), no fertilizer (NF), cross-pollination (CP) and self-pollination (SP). The content of a wide range of nutrients (listed in Table 2) was quantified in the laboratory (methods detailed in the supporting information S1). Permutational multivariate analysis of variance using Bray-Curtis distances was performed on the nutritional content of the almonds, with the treatment as the grouping factor, replicated at the tree level. Each point on the graph represents an individual tree that was exposed to one of the treatments and the hulls link trees exposed to the same treatment. The further apart the points are on the graph, the more different the nuts’ nutritional composition.

The overall nutritional composition of the almonds differed between treatment groups (Fig. 1, stress 10%). This was confirmed by the permutational multivariate analysis of variance, which found that 36% of the difference in nutritional composition was related to the treatment group (R^2^ = 0.36, *F*_3_ = 2.7, *P* = 0.026). The variation in nutritional composition did not differ between treatment groups (*F*_3_ = 1.3, *P* = 0.339).

**Figure 2:**
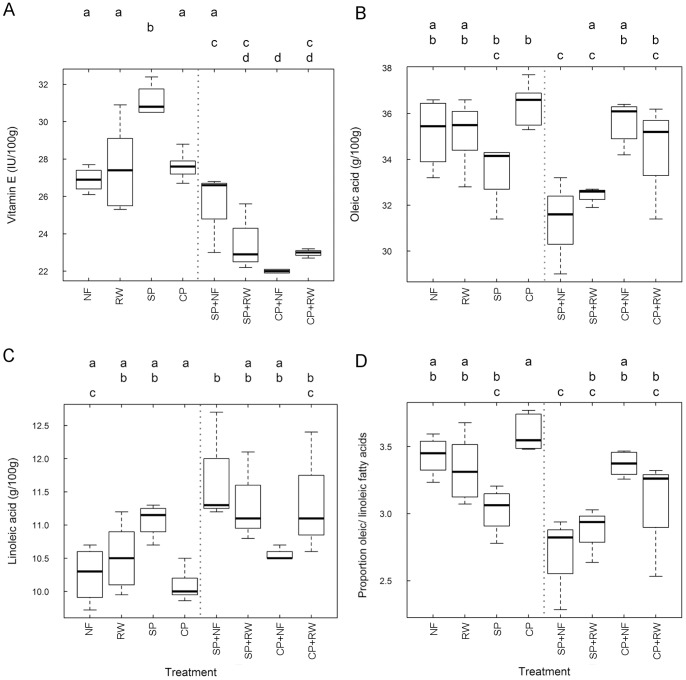
The nutritional composition of almonds from trees that received different resource treatments alone and in combination. Treatments: no fertilizer (NF), reduced water (RW), self-pollination (SP) and cross-pollination (CP). Single and combined treatments are separated by a dashed line. The boxes show the median, 25^th^ and 75^th^ percentiles and the letters a,b,c,d, indicate significant differences from Tukey’s HSD test at *P*<0.05.

We found differences between the eight treatment combinations in the levels of vitamin E (F_7_ = 20.4, *P*<0.001), oleic acid (F_7_ = 5.0, *P* = 0.001) and linoleic acid (F_7_ = 5.0, *P* = 0.001). Vitamin E was highest in the self-pollinated nuts and was lowest in the nuts from treatments combined with hand cross-pollination, especially cross-pollinated with no fertilizer (Fig. 2a). However, oleic acid was lower in the self-pollinated nuts of the single treatments (Fig. 2b), and lower still in self-pollinated nuts combined with reduced water and no fertilizer. Linoleic acid was highest in the self-pollinated nuts combined with no fertilizer and lowest in nuts from cross-pollinated trees (Fig. 2c). The proportion oleic to linoleic acid (F_7_ = 7.1, *P*<0.001) varied between treatments (Fig. 2d). It was lower in self-pollinated nuts and decreased further when self-pollination was combined with no fertilizer.

## Discussion

While the adverse effects of pollination limitation and resource scarcity on fruit set and crop development have been documented [15], [17], [28], this is the first study to our knowledge, to test if pollination and plant resources act alone or in combination on the nutritional composition of a crop. The effect of self-pollination on the nutritional composition of Nonpareil almonds was greater than the effect of reduced water and fertilizer. The health benefits of almonds are attributed to their fat composition and vitamin E, and these were both affected by the pollination treatments.

We found the highest oleic to linoleic ratio in almonds in cross-pollinated trees, and the lowest ratio in pollinator-excluded trees. Almonds with a high oleic to linoleic ratio would be most favorable to consumers seeking health benefits. The high oleic acid content is credited for the cardio-protective effect of almonds [19], [29]. Although linoleic acid is an essential fatty acid that the body cannot synthesize, rising dietary levels of omega-6 fatty acids are believed to contribute to chronic diseases in Western cultures [21]. In terms of crop characteristics, a high oleic (mono-unsaturated fatty acid) to linoleic (an omega-6 polyunsaturated fat) acid ratio is desirable as it improves the stability of the fats against rancidity and therefore increases the almonds’ shelf life [27]. A positive effect of bee-pollinated flowers on shelf life was recently demonstrated for strawberry production, since bee pollination leads to firmer fruits that last longer [8]. Further, Klatt *et al*. [8] show that bee pollination increased fruit weight and redness and also reduced sugar-acid ratios and therefore produced higher commercial grades. Together these two effects increased the value attributed to bee-pollinated fruits. Other studies showed that oil content in oilseed rape (*Brassica napus* var. SW Stratos™) [30] and sugar content in mandarin orange (*Citrus reticulata* Blanco) [31] were improved by pollination. Negative effects of limited pollination have included lowered calcium concentrations (associated with storage disorders) in Braeburn apples (*Malus domestica* Borkh) [32], although this effect seems to be variety specific [9] and lowered fruit weight and length in self-pollinated *Cucurbita moschata*, cv. Piramoita [33]. Consistent with previous studies, our overall findings were that cross-pollinated trees bore fruit with the most favorable nutritional content. A notable exception in our study was vitamin E (α Tocopherol), which was higher in self-pollinated almonds.

The mechanism(s) for the impact of cross- *versus* self-pollination on nutrients is as yet unknown. Self-pollination in almond may alter the nutritional composition of the nuts through a reduced development rate. Higher levels of linoleic acid are found in the earlier stages of oil accumulation in almond, with levels declining in the later developmental stages as oleic acid increases [34]. The self-pollinated almonds may develop more slowly than the cross-pollinated almonds. Compatible pollen tubes were found to grow more quickly in almond than self-pollen tubes, and reached the ovary earlier [35]. In addition, self-pollination was found to delay megasporocyte differentiation [36]. If self-pollinated almonds develop more slowly, at harvest time they may not have accumulated as much oleic acid or lost as much linoleic acid as cross-pollinated almonds. Given that self-pollen tubes grow more slowly [35], in self-pollinated flowers there may also be an effect of embryo sac degeneration on the fruit’s development and nutritional composition. While the mesh cages used to exclude pollinators from the trees could also have played a role in slowing fruit development by reducing the amount of sunlight the trees received, we would expect this to be minor due to the relatively short length of time the cages were covering the trees. Klatt *et al*. [8] explained their findings of increased strawberry shelf life with bee pollination as deriving from the increased amount of fertilized achenes per fruit (strawberry is an aggregate accessory fruit, consisting of many achenes each containing a single seed) when pollinated by bees. These achenes both create firmness structurally and produce the plant hormones that prevent fruit softening. It remains to be tested for any kind of crop if plant hormone production differs in self- versus cross-pollinated fruits or seeds.

In contrast to the pollination treatment, the fertilizer and water treatments had the potential to alter the resources available to the trees after the flowers had finished blooming. However, the limited impact of the fertilizer regime on almond’s nutritional composition suggests that sufficient resources may have been stored in the soil from prior fertilizations. An effect of reduced resources such as fertilizer on almond composition may only be detected after multiple years with no fertilizer; especially as perennial almond trees may also store resources [17]. Reductions in irrigation have been found to reduce almond production and yield during the drought year [23] and in subsequent years [37]. The lack of impact of reduced water on the nutritional content of the almonds is supported by a review of almond composition studies, which found that irrigation had little effect on the oil content [38].

We did not analyze nutrients of almonds from open-(bee) pollinated trees not treated with reduced water or no fertilizer in the same study year. In a previous study [23], Klein *et al*. investigated the weight and number of nuts produced by these same almond trees as well as open-pollinated trees and found that self-pollinated trees produced the fewest, heaviest nuts. Open pollinated nuts were intermediate with respect to weight and number, meaning that hand cross-pollinated trees produced larger numbers of smaller, lighter nuts. Nut weight and number was strongly related to the pollination treatments and the plant resource treatments had little influence [23]. This led us to hypothesize that the effect of self- versus cross-pollinated nuts could be indicated by nut size. We therefore analyzed the same nutrients for small and large open-pollinated nuts collected randomly in the experimental orchard under normal water and fertilizer regimes a year after the experiment took place. However, contrary to our expectations we found that the nutrient content of large and small nuts were not significantly different (data not shown). We conclude that although pollination can influence fruit weight, the weight of a fruit seems not to influence the amount of nutrients per unit weight in the nuts. Since these two processes are decoupled, this suggests that pollination directly influences the ratio of these nutrients that are important for human health.

The nutritional composition of crops may vary under a variety of interacting factors including agricultural practices, pollination and climate. The combined resource manipulations in this study showed little interactive effect on the fat composition. For vitamin E however, the combined resource and pollination treatments showed different levels of vitamin E than would have been expected from the effects of the single treatments. This suggests that changes in pollination could have unexpected effects on crop nutrition due to interactions with other inputs. In tomato (*Lycopersicon esculentum* L.), the benefits of arbuscular mycorrhizal fungal colonization for fruit quality (lower acidity, greater ascobrbic acid content) were found to be more pronounced when the plants were water stressed [39].

The present study demonstrates that pollination can positively change the nutritional value and commercial quality of almonds and that the effects are even stronger in combination with reduced availability of water and especially of nutrients applied to the soil. The potential nutritional benefits from cross-pollination deserve further study and may increase the current estimates of the economic and health value of pollination service to crops [4], [8], [40], [41].

## Supporting Information

Supporting information S1 Details of the methods used for the nutritional analysis of almonds.
(DOCX)

Supporting information S2 The results of analyses of variance of the nutrients in almonds (listed in Table 2) from trees exposed to a single resource or pollination treatment.
(DOCX)

Supporting information S3 A correlation matrix of the different nutrients quantified in almonds.
(DOCX)
