# Supplementary material for: Pollination and Plant Resources Change the Nutritional Quality of Almonds for Human Health
Source: PLoS One. 2014 Feb 27;9(2):e90082. doi: 10.1371/journal.pone.0090082 (PMC3937406; doi:10.1371/journal.pone.0090082)
Supplement: Supporting information S1 — Details of the methods used for the nutritional analysis of almonds. (DOCX) [file pone.0090082.s001.docx]

**Supporting information S1** Details of the methods used for the nutritional analysis of almonds. All nutritional analyses were performed by NP Analytical Laboratories, St. Louis, MO, U.S.A. and follow the Official Methods of Analysis of the Association of Analytical Communities (AOAC). References relating to the methods are given in italics.

**Fat Composition**

Oil was extracted from the almond samples by soxhlet extraction. The extracted oil was saponified with alcoholic sodium hydroxide. The fatty acids were esterified in methanol, with boron trifluoride as a catalyst, taken up in heptanes, and injected on a gas chromatograph with a flame ionization detector. The fatty acid methyl esters were calculated from a set of standards containing known concentrations of prepared methyl esters of selected fatty acids. The concentration of each fatty acid methyl ester was converted by calculation to either equivalent triglyceride or fatty acid.

*Official Methods of Analysis of the AOAC International, 17th Edition, Method 996.06, 983.23, Method 969.33.*

**Vitamin E**

The samples and a standard were saponified with an alcoholic KOH solution in the presence of an antioxidant (pyrogallol) to convert tocopherol esters to the alcohol (tocopherol). An aliquot of the extract was shaken with hexane, phasing the tocopherol into hexane. An aliquot of hexane was evaporated and the extract reconstituted in methanol. The reconstituted extract was injected on a High Pressure Liquid Chromatograph (HPLC) equipped with a flouresence detector for vitamin E analysis. Vitamin E was quantified from a set of standard solutions of known concentration that were taken through the method.

*Official Methods of Analysis of AOACI, Methods 992.06 and 2001.13 (modified).*

**Vitamin B1**

Free thiamine (vitamin B1) was extracted by autoclaving with dilute hydrochloric acid solution. Bound thiamine was released by incubating overnight in an enzyme solution. Excess protein was precipitated by a pH adjustment and filtration. Thiamine was phased into isobutyl alcohol to remove fluorescent interferences. The thiamine extract was oxidized with alkaline potassium ferricyanide to form thiochrome which was measure fluorometrically and quantified from a set of standards of known concentration that were taken through the procedure from the oxidation step.

*Official Methods of Analysis of the AOAC, (2000) 17th Ed., Method 942.23, Locator #45.1.05 (modified).*

**Vitamin B2**

Riboflavin (vitamin B2) was extracted with a urea-acid mixture, diluted and filtered. Samples and standards were measured fluorometrically using an Alpkem RFA-300 system. Blank readings were determined by reducing riboflavin to a non-fluorescent compound using sodium hydrosulfite and reading any background fluorescence. Riboflavin concentration was quantified from a set of standards of known concentration.

*Official Methods of Analysis of the AOAC International, 17th Edition, Method 981.15 Locator #45.1.09.*

**Vitamin B3**

A turbidimetric microbiological method was used based on growth of *Lactobacillus planatarum* ATCC 8014, which requires niacin (vitamin B3) for growth. A basal medium, nutritionally complete in all respects except for niacin, was used as the dilutent for the final dilutions of the standards and samples. Following incubation, the growth response of the bacterial cultures was measured as percent transmittance. A dose-response line was constructed and the sample concentrations calculated.

*Official Methods of Analysis of the AOAC, (1995), Method 960.46.*

**Minerals**

The sample was prepared via a dry ashing procedure to remove organic material. The ash residue was dissolved in dilute acid. The concentrations of the various minerals were determined by comparing the characteristic emission spectra of each sample to the emissions spectra of a series of standard solutions as measured by ICP spectroscopy.

*Official Methods of Analysis of the AOAC International, Method 968.08, Method 984.27, Method 935.13, Method 985.35, Methods 975.03, Method 990.08, and Method 993.14.*

**Sugars**

Sugars were extracted from the sample with warm alcohol-water mixture. Sample extracts were injected on a high pressure liquid chromatograph (HPLC) with a refractive index detector, and quantitated from a set of standards of know concentration also injected on the HPLC.

*AACC International Approved Methods, 80-04, (modified).*
