# Supplementary material for: Pollination and Plant Resources Change the Nutritional Quality of Almonds for Human Health
Source: PLoS One. 2014 Feb 27;9(2):e90082. doi: 10.1371/journal.pone.0090082 (PMC3937406; doi:10.1371/journal.pone.0090082)
Supplement: Supporting information S2 — The results of analyses of variance of the nutrients in almonds (listed in Table 2) from trees exposed to a single resource or pollination treatment. (DOCX) [file pone.0090082.s002.docx]

**Supporting information S2** The results of analyses of variance of the nutrients in almonds (listed in Table 2) from trees exposed to single resource or pollination treatments (only the nutrients that differed between treatments are listed (mean±SE), the overall significance of treatment is represented by **P≤*0.05, ** *P*<0.01 and *** *P*<0.001 (3 d.f.)).

| **Nutrient** | **Cross-pollination**  **(CP)** | **Self-pollination**  **(SP)** | **No**  **fertilizer**  **(NF)** | **Reduced water**  **(RW)** | **Pairwise**  **comparison^#^** |
| --- | --- | --- | --- | --- | --- |
| Oleic fatty acid* (g/100g) | 36.4  ±0.6 | 33.5  ±1.0 | 35.2  ±1.0 | 35.1  ±0.9 | CP>SP |
| Linoleic fatty acid*  (g/100g) | 10.1  ±0.2 | 11.1  ±0.3 | 10.4  ±0.3 | 10.5  ±0.2 | SP>CP,NF |
| **^¶^**Proportion Oleic/Linoleic** | 3.6  ±0.1 | 3.0  ±0.1 | 3.4  ±0.1 | 3.3  ±0.1 | CP,NF>SP |
| Phosphorous*  (ppm) | 5618  ±115 | 6018  ±172 | 5594  ±172 | 5390  ±163 | SP>RW |
| Potassium*  (ppm) | 6977  ±165 | 7758  ±247 | 7041  ±247 | 7019  ±233 | SP>CP,RW |
| Fructose*  (%) | 0.130  ±0.003 | 0.123  ±0.005 | 0.123  ±0.005 | 0.112  ±0.005 | CP>RW |
| Glucose*  (%) | 0.200  ±0.003 | 0.188  ±0.005 | 0.198  ±0.005 | 0.206  ±0.004 | RW>SP |
| Vitamin E** (IU/100g) | 27.6  ±0.6 | 31.1  ±1.0 | 26.9  ±1.0 | 27.6  ±0.9 | SP>CP,NF,RW |

**^¶^**The proportion of oleic to linoleic fatty acids was tested, as this is an indicator of almond quality.

**^#^**The direction of significant (*P*≤0.05) pairwise relationships is indicated from the results of Tukey’s HSD tests.
