# Supplementary material for: Pollination and Plant Resources Change the Nutritional Quality of Almonds for Human Health
Source: PLoS One. 2014 Feb 27;9(2):e90082. doi: 10.1371/journal.pone.0090082 (PMC3937406; doi:10.1371/journal.pone.0090082)
Supplement: Supporting information S3 — A correlation matrix of the different nutrients quantified in almonds. (DOCX) [file pone.0090082.s003.docx]

**Supporting information S3** A correlation matrix of the different nutrients quantified in almonds. Significant correlations are given in bold (table continues on next page).

|  | Thiamine | Riboflavin | Oleic | Linoleic | Monounsat | Polyunsat | Saturated | Total Fat | Calcium | Copper |
| --- | --- | --- | --- | --- | --- | --- | --- | --- | --- | --- |
| Riboflavin | -0.07 | - | - | - | - | - | - | - | - | - |
| Oleic | -0.12 | -0.23 | - | - | - | - | - | - | - | - |
| Linoleic | **0.47** | 0.12 | **-0.51** | - | - | - | - | - | - | - |
| Monounsat | -0.11 | -0.29 | **0.99** | **-0.47** | - | - | - | - | - | - |
| Polyunsat | 0.43 | 0.12 | **-0.49** | **1.00** | -0.46 | - | - | - | - | - |
| Saturated | 0.21 | -0.08 | 0.41 | 0.42 | 0.42 | 0.41 | - | - | - | - |
| Total Fat | 0.07 | -0.20 | **0.92** | -0.13 | **0.92** | -0.12 | **0.67** | - | - | - |
| Calcium | 0.26 | **-0.49** | 0.20 | -0.28 | 0.17 | -0.29 | -0.05 | 0.11 | - | - |
| Copper | -0.30 | 0.05 | 0.00 | -0.03 | 0.02 | 0.01 | -0.32 | -0.04 | -0.28 | - |
| Iron | -0.15 | -0.05 | -0.09 | -0.43 | -0.11 | -0.41 | **-0.70** | -0.31 | 0.36 | **0.56** |
| Magnesium | 0.45 | -0.02 | -0.07 | 0.35 | -0.05 | 0.37 | 0.31 | 0.10 | 0.26 | -0.03 |
| Manganese | -0.12 | -0.38 | -0.01 | -0.35 | -0.02 | -0.36 | -0.42 | -0.17 | 0.34 | 0.45 |
| Phosphorus | -0.05 | 0.30 | -0.45 | 0.16 | -0.43 | 0.17 | -0.38 | -0.44 | -0.28 | **0.49** |
| Potassium | -0.03 | 0.30 | **-0.62** | **0.51** | **-0.64** | **0.52** | -0.06 | **-0.48** | -0.41 | 0.28 |
| Sodium | 0.40 | 0.41 | -0.38 | 0.43 | -0.39 | 0.40 | 0.17 | -0.24 | -0.07 | -0.14 |
| Zinc | **-0.53** | 0.39 | -0.38 | -0.13 | -0.39 | -0.10 | **-0.63** | **-0.50** | -0.30 | **0.64** |
| Niacin | **0.75** | -0.20 | 0.20 | 0.25 | 0.19 | 0.22 | **0.52** | 0.35 | 0.37 | -0.53 |
| Fructose | -0.33 | 0.04 | 0.19 | 0.09 | 0.24 | 0.11 | 0.35 | 0.29 | -0.21 | 0.14 |
| Glucose | 0.09 | -0.45 | **0.64** | -0.26 | **0.64** | -0.27 | 0.42 | **0.61** | 0.25 | -0.23 |
| Sucrose | **0.53** | 0.11 | -0.14 | **0.78** | -0.13 | **0.77** | **0.58** | 0.19 | -0.02 | -0.06 |
| Vitamin E | 0.41 | 0.41 | -0.39 | **0.64** | -0.41 | **0.66** | 0.24 | -0.14 | -0.04 | -0.10 |

**Supporting information S3** continued

|  | Iron | Magnesium | Manganese | Phosphorus | Potassium | Sodium | Zinc | Niacin | Fructose | Glucose | Sucrose |
| --- | --- | --- | --- | --- | --- | --- | --- | --- | --- | --- | --- |
| Magnesium | 0.14 | - | - | - | - | - | - | - | - | - | - |
| Manganese | **0.61** | -0.12 | - | - | - | - | - | - | - | - | - |
| Phosphorus | 0.42 | 0.20 | 0.34 | - | - | - | - | - | - | - | - |
| Potassium | 0.00 | 0.18 | 0.20 | **0.72** | - | - | - | - | - | - | - |
| Sodium | -0.22 | 0.31 | -0.35 | -0.11 | 0.14 | - | - | - | - | - | - |
| Zinc | **0.56** | -0.22 | 0.33 | **0.74** | 0.46 | -0.20 | - | - | - | - | - |
| Niacin | -0.34 | **0.57** | -0.25 | -0.43 | -0.20 | 0.34 | **-0.82** | - | - | - | - |
| Fructose | -0.03 | 0.03 | -0.05 | 0.08 | -0.02 | -0.31 | 0.08 | -0.22 | - | - | - |
| Glucose | -0.36 | -0.02 | -0.08 | **-0.70** | -0.59 | 0.05 | **-0.70** | 0.38 | -0.23 | - | - |
| Sucrose | **-0.43** | 0.46 | -0.43 | -0.09 | 0.15 | **0.59** | -0.35 | 0.42 | -0.03 | 0.19 | - |
| Vitamin E | -0.17 | 0.39 | -0.21 | 0.33 | **0.60** | 0.35 | 0.01 | 0.27 | 0.00 | -0.37 | **0.52** |
